# Identification of high‐performance blood‐based biomarkers for early screening and staging of Alzheimer’s disease by large‐scale plasma proteomic profiling in mild cognitive impairments

**DOI:** 10.1002/alz.086052

**Published:** 2025-01-09

**Authors:** Yuanbing JIANG, Hyebin Uhm, Fanny C. F. Ip, Ronnie M.N. Lo, Xiaoyun Cao, Clara M.C. Tan, Brian C.H. Law, Timothy CY Kwok, Vincent Chung Tong Mok, Henrik Zetterberg, Amy K.Y. Fu, Nancy Y. Ip

**Affiliations:** ^1^ Division of Life Science, State Key Laboratory of Molecular Neuroscience, Molecular Neuroscience Center, The Hong Kong University of Science and Technology, Clear Water Bay, Kowloon, Hong Kong China; ^2^ Hong Kong Center for Neurodegenerative Diseases, Hong Kong Science Park, Hong Kong China; ^3^ Guangdong Provincial Key Laboratory of Brain Science, Disease and Drug Development, HKUST Shenzhen Research Institute, Shenzhen–Hong Kong Institute of Brain Science, Shenzhen, Guangdong, 518057 China; ^4^ Therese Pei Fong Chow Research Centre for Prevention of Dementia, Division of Geriatrics, Department of Medicine and Therapeutics, The Chinese University of Hong Kong, Shatin, Hong Kong China; ^5^ Lau Tat‐chuen Research Centre of Brain Degenerative Diseases in Chinese, Gerald Choa Neuroscience Institute, Therese Pei Fong Chow Research Centre for Prevention of Dementia, The Chinese University of Hong Kong, Prince of Wales Hospital, Hong Kong SAR Hong Kong; ^6^ Hong Kong Center for Neurodegenerative Diseases, Hong Kong China; ^7^ Department of Neurodegenerative Disease, UCL Queen Square Institute of Neurology, University College London, London, ‐ UK; ^8^ Wisconsin Alzheimer's Disease Research Center, School of Medicine and Public Health, University of Wisconsin‐Madison, Madison, WI USA; ^9^ UK Dementia Research Institute at UCL, London UK; ^10^ Department of Psychiatry and Neurochemistry, Institute of Neuroscience and Physiology, the Sahlgrenska Academy at the University of Gothenburg, Mölndal Sweden; ^11^ Department of Psychiatry and Neurochemistry, Institute of Neuroscience and Physiology, The Sahlgrenska Academy at the University of Gothenburg, Mölndal Sweden

## Abstract

**Background:**

Early detection of Alzheimer's disease (AD), particularly during the mild cognitive impairment (MCI) stage, is crucial for effective intervention and treatment. However, existing diagnostic methods, through cognitive assessments, brain imaging, or cerebrospinal fluid profiling, are subjective, expensive or invasive. We previously showed that the plasma proteome is altered in AD, suggesting the feasibility of utilizing blood biomarkers for AD detection. Therefore, to develop a blood test for early screening and staging of AD, it is important to identify blood biomarkers associated with MCI/early AD and the features of AD progression.

**Method:**

We conducted a comprehensive profiling of the plasma proteome of MCI by measuring 1,160 proteins in a Hong Kong Chinese cohort, to identify novel blood biomarkers for MCI/early AD. We further developed a biomarker panel based on the identified MCI/AD‐associated blood biomarkers and validated its performance in an independent amyloid‐PET‐defined cohort.

**Result:**

We identified 496 proteins that were dysregulated in MCI plasma, and these proteins, involved in different biological processes, exhibited distinct dysregulating patterns with disease progression. We further categorized these proteins into different groups based on their dysregulating patterns. By performing co‐expression network analysis, we identified an 18‐protein panel that captures the profile changes of plasma proteome in MCI and AD. This panel accurately classified MCI (AUC = 0.913‐0.925) and AD (AUC = 0.970‐0.993) in two independent cohorts. Moreover, this panel correlates with cognitive decline and the development of Aβ pathology in the brain, which outperforms some of the existing plasma ATN biomarkers.

**Conclusion:**

This study comprehensively profiled the MCI plasma proteome and demonstrates the feasibility of a blood‐based biomarker assay for early detection and staging of MCI and AD, potentially facilitating early screening, monitoring, and intervention in future clinical settings.

